# The role of mtDAMPs in the trauma-induced systemic inflammatory response syndrome

**DOI:** 10.3389/fimmu.2023.1164187

**Published:** 2023-07-18

**Authors:** Jingjing Ye, Xiaodan Hu, Zhiwei Wang, Rui Li, Lebin Gan, Mengwei Zhang, Tianbing Wang

**Affiliations:** ^1^ Trauma Center, Peking University People’s Hospital, Key Laboratory of Trauma Treatment and Neural Regeneration (Peking University) Ministry of Education, National Center for Trauma Medicine of China, Beijing, China; ^2^ School of Basic Medicine, Peking University, Beijing, China; ^3^ Orthopedics Department, The First Affiliated Hospital of Zhengzhou University, Zhengzhou, China

**Keywords:** mtDAMPs, trauma-induced SIRS, mtDNA, mtFPs, trauma

## Abstract

Systemic inflammatory response syndrome (SIRS) is a non-specific exaggerated defense response caused by infectious or non-infectious stressors such as trauma, burn, surgery, ischemia and reperfusion, and malignancy, which can eventually lead to an uncontrolled inflammatory response. In addition to the early mortality due to the “first hits” after trauma, the trauma-induced SIRS and multiple organ dysfunction syndrome (MODS) are the main reasons for the poor prognosis of trauma patients as “second hits”. Unlike infection-induced SIRS caused by pathogen-associated molecular patterns (PAMPs), trauma-induced SIRS is mainly mediated by damage-associated molecular patterns (DAMPs) including mitochondrial DAMPs (mtDAMPs). MtDAMPs released after trauma-induced mitochondrial injury, including mitochondrial DNA (mtDNA) and mitochondrial formyl peptides (mtFPs), can activate inflammatory response through multiple inflammatory signaling pathways. This review summarizes the role and mechanism of mtDAMPs in the occurrence and development of trauma-induced SIRS.

## Introduction

Systemic inflammatory response syndrome (SIRS) is a non-specific exaggerated defense response caused by infectious or non-infectious stressors such as trauma, burn, surgery, ischemia and reperfusion, and malignancy, which can eventually lead to an uncontrolled inflammatory response (1). In trauma medicine, SIRS can be regarded as an independent predictor of mortality after trauma (2). Moreover, trauma-induced SIRS increases susceptibility to infections, which can lead to multiple organ dysfunction syndrome (MODS), which is often detrimental and leads to poor prognosis in trauma patients (3). Therefore, understanding the role and mechanism of trauma-induced SIRS may provide new perspectives for clinical diagnosis, treatment, and scientific research.

Early studies indicate that trauma-induced tissue damage leads to pathogen invasion and the release of pathogen-associated molecular patterns (PAMPs). The binding of specific pattern recognition receptors (PRRs) by PAMPs activates the innate immune system, a prerequisite step for generating immunogenic signals that ultimately lead to infectious SIRS (4). Gradually, researchers found that SIRS is not caused by pathogens (5) but rather by the endogenous damage-associated molecular patterns (DAMPs) released by tissue injury in many trauma patients. Just like PAMPs, DAMPs can bind to PRRs, activate the immune system, and cause further tissue damage. It is largely accepted that DAMPs can be sensed by several classical PRRs, including Toll-like receptors (TLRs), C-type lectin receptors (CLRs), retinoic acid-inducible gene (RIG)-I-like receptors (RLRs), NOD-like receptors (NLRs), and multiple intracellular DNA sensors (6–8). DAMPs are mainly derived from the plasma membrane, nucleus, cytosol, and mitochondria (9). Mitochondrial DAMPs (mtDAMPs) mainly include mitochondrial DNA (mtDNA), mitochondrial formyl peptides (mtFPs), mitochondrial transcription factor A (TFAM), cardiolipin (CL), ATP, cytochrome *c*, and mitochondrial RNA (mtRNA), which are also related to trauma-induced SIRS (5).

Trauma causes mitochondrial damage and dysfunction, leading to mtDAMP release and the induction of an immune response similar to that against pathogen infection (10). This phenomenon is theoretically supported by the danger model of immune recognition and the endosymbiotic theory. Matzinger’s danger model theory points out that the activation of the body’s immune system occurs through the recognition of danger signals released by damaged tissues rather than by the recognition of non-self-molecules (11). These endogenous danger signals are called DAMPs, which normally avoid contact with the immune system because of the plasma membrane and intracellular compartmentalization (12). When cells and tissues are damaged, DAMPs are released into the extracellular space or circulation (12). According to the endosymbiotic theory, the mitochondria evolved from ancient bacterial endosymbiont; therefore, the endogenous molecules released by the mitochondria are similar to bacterial PAMPs, which mediate inflammatory reactions similar to bacterial infections. This review focuses on the research progress of mtDAMPs in trauma-induced SIRS and summarizes the development and pathophysiology at the cellular and organ levels.

## Cellular level study of mtDAMPs in trauma-induced SIRS

### MtDNA

Human mtDNA is a closed-circular double-stranded molecule coding 37 genes. Unlike nuclear DNA, mtDNA can be easily damaged by the lack of repair systems and histone protection (13). Similar to bacterial DNA, mtDNA contains unmethylated CpG repeats that are recognized by the immune system as non-self. Post-traumatic mitochondrial damage causes mtDNA released outside the mitochondria (10, 14, 15). A recent study in a trauma cohort from Briggs GD showed that the size of mtDNA in the circulation post-trauma is a mixture of “larger forms” and low molecular weight mtDNA, and it was the low-molecular-weight version of cell-free mtDNA that is associated with inflammation and poor clinical outcomes post-trauma (16). Free mtDNA mediates inflammation through a variety of signaling pathways (17).

#### Toll-like receptor 9 signaling pathway

As a PRR, Toll-like receptor 9 (TLR9) can directly bind to CpG DNA. After the stimulation by CpG DNA, TLR9 and its intracellular adaptor protein myeloid differentiation factor 88 (MyD88) localize in the endoplasmic reticulum, rapidly redistribute to the CpG DNA accumulation site, and transfer to the endosomal membrane and lysosomal compartment (18).

MtDNA/TLR9 signaling pathway has been demonstrated in various cell types, such as dendritic cells, neutrophils, macrophages, and natural killer cells (19). Stimulated TLR9 binds to MyD88 to activate the nuclear factor kappa-B (NF-κB) and mitogen-activated protein kinase (MAPK) cascades (6). The activation of NF-κB induces the expression of tumor necrosis factor-α (TNF-α), interleukin-1β (IL-1β), interleukin-6 (IL-6), and other proinflammatory cytokines (6). The activation of MAPK cascades induces activator protein-1 (AP-1) formation and promotes cytokine expression (6). In plasmacytoid dendritic cells, TLR9/MyD88 activates interferon regulatory factor 7 (IRF7), which transfers to the nucleus, and induces the expression of type I interferon (20). In contrast, in myeloid dendritic cells and macrophages, TLR9/MyD88 activates IRF1 and induces the expression of interferon-β (IFN-β) (21). In addition, mtDNA activates neutrophils through TLR9, causing Ca^2+^ influx and MAPK phosphorylation, mediating neutrophil degranulation and migration, and inducing inflammation (10).

#### Cyclic GMP-AMP synthase/interferon gene stimulator signaling pathway

Cyclic GMP-AMP synthase (cGAS) and interferon gene stimulator (STING) are widely expressed in mammalian cells that mediate the expression of type I interferon and other cytokines in infections and inflammatory diseases (22). cGAS is mainly limited in the cytoplasm to avoid the continuous activation of its DNA in the nucleus. However, recent studies have demonstrated that cGAS also exists in the nucleus (23) and plasma membrane (24).

cGAS binds to mtDNA and catalyzes the generation of 2′-3′ cyclic GMP-AMP (cGAMP) from its substrates GTP and AMP (25). The secondary messenger cGAMP binds to STING in the endoplasmic reticulum (ER) membrane, thereby promoting STING conformational change and forming dimers (26). The activated STING is transferred to the ER–Golgi intermediates and Golgi by vesicle transport (27). In this process, STING triggers inflammatory storms and interferes with autophagy in an interferon-dependent manner (28). There are two main downstream pathways of STING (29, 30). In the first pathway, STING directly binds and phosphorylates TANK-binding kinase 1 (TBK1), mediates the phosphorylation and nuclear transfer of IRF3, and initiates the expression of type I IFN. In the second pathway, STING directly binds and phosphorylates the inhibitor of κB kinase (IKK) complex, activates NF-κB, and promotes the expression of cytokines, such as IL-1β, IL-6, and TNF-α.

#### NLRP3 inflammasomes

Nucleotide oligomerization domain-like receptor thermal protein domain associated protein 3 (NLRP3) inflammasome is a macromolecular complex composed of NLRP3, caspase-1, and apoptosis-associated speck-like protein containing a caspase recruitment domain (ASC) (31). It is usually present in neutrophils, monocytes, dendritic cells, macrophages, and non-hematopoietic cells (32). A study indicated that NLRP3 inflammasomes could be activated through three different signaling pathways: canonical NLRP3 activation, non-canonical NLRP3 activation, and alternative NLRP2 activation (33). The typical NLRP3 activation pathway involves two steps, namely, initiation and activation. The initiation step revolves around TLR recognition of PAMP or DAMP, activation of NF-κB, synthesis of IL-1β and IL-18 precursor, and expression of NLRP3 (34). However, the exact mechanism of the activation step remains unclear. Several NLRP3 agonists trigger the activation of NLRP3 inflammasome; however, these agonists are not chemically or structurally related, and there is little evidence regarding whether NLRP3 directly binds them (33). A possible explanation is that NLRP3 agonist-mediated molecular and cellular events (including K^+^ outflow, mitochondrial dysfunction, reactive oxygen species (ROS) and mtDNA release, and lysosomal destruction) are upstream signals for inflammasome assembly and activation (35, 36). However, these events do not apply to all NLRP3 agonists, and a consistent NLRP3 activation pathway is lacking (35).

Unlike cGAS, which binds to both oxidized and non-oxidized mtDNAs, NLRP3 prefers oxidized mtDNA (ox-mtDNA) (13, 37). Ox-mtDNA exits the mitochondria through mitochondrial permeability transition pores and voltage-dependent ion channels and acts as a ligand to activate the NLRP3 inflammasome (38). Extracellular mtDNA does not serve as an NLRP3 ligand directly, while it triggers the TLR9/MAPK/NF-κB pathway to induce the initiation and activation of NLRP3 (39). After NLRP3 activation, the activated caspase-1 processes IL-1β and IL-18 precursor into a mature secreted form. Moreover, it cleaves gasdermin D (GSDMD) that is subsequently transferred to the cell membrane to form pores, thereby mediating the release of proinflammatory cytokines and causing pyroptosis (40). In addition, gasdermin D promotes mitochondrial fragmentation and mtDNA release (41).

Zhong et al. confirmed that the newly synthesized mtDNA is indispensable for NLRP3 inflammasome activation (42). TLR activation triggers the IRF1-dependent transcription of CMPK2, which acts as a rate-limiting enzyme to catalyze mtDNA synthesis after exposure to NLRP3 agonists (42). The newly synthesized mtDNA is oxidized by mitochondrial ROS to generate ox-mtDNA, which subsequently triggers NLRP3 inflammasome activation (42). Xian et al. stimulated mice with myeloid-specific knockout of cytidine/uridine monophosphate kinase (CMPK2) with lipopolysaccharide (LPS) and observed decreased NLRP3 inflammasome activation and blocked IL-1β secretion in mouse alveolar macrophages, which is consistent with the results of Zhong et al. (42, 43). However, in the study on severe fever caused by thrombocytopenia syndrome virus infection, ox-mtDNA activated NLRP3 inflammasome, but no significant new mtDNA synthesis was observed (44). Therefore, whether newly synthesized mtDNA is necessary for NLRP3 inflammasome activation remains unclear.

### MtFPs

MtFPs are proteins synthesized from mtDNA, and like in bacteria, *N*-formyl methionine is the first amino acid during translation initiation. Severe trauma causes mitochondrial damage and the release of mtFPs into the blood circulation, where they bind and activate formyl peptide receptor (FPR), and recruit immune cells to mediate inflammatory response (45). FPR1, FPR2, and FPR3 are three human FPRs (5) expressed in multiple cell types. The highest expression levels of FPR1 and FPR2 have been detected in neutrophils, and those of FPR3 have been detected in mononuclear macrophages (46). Among FPR ligands, mtFPs are the only ligand common to all human FPRs (46).

Downstream signaling pathways after FPR activation remain unclear. Hazeldine et al. demonstrated that mtFPs activate neutrophils through extracellular signal-related kinase 1/2 (ERK1/2) and MAPK signaling pathways (47). MtFP-stimulated FPR1 induces chemotaxis, degranulation, and Ca^2+^ outflow of the polymorphonuclear neutrophils (PMNs); promotes ROS and proinflammatory cytokine production; and enhances cytoskeletal rearrangement and network formation (5, 48). Liu et al. discovered that FPR2 can directly interact with transforming growth factor-β-activated kinase 1 (TAK1), thereby enhancing inflammation and oxidative stress related to Nrf2 activation (49). Lee et al. confirmed the presence of FPR3 in neutrophils; moreover, the use of FPR3 agonists could activate neutrophils, inhibit inflammatory cytokines generation, and kill bacteria through ROS generation (50). Studies have discovered that the binding of mtFPs to PMN stimulating FPR1 leads to the desensitization and inline of multiple PMN chemokine receptors, thereby reducing the number of PMNs that can migrate to secondary infection sites (51, 52). Therefore, the blockage of FPR1 not only protects the receptors from desensitization but also preserves the immune response at the injection site (triggered by the stimulation of monocytes with PAMP), which improves overall anti-pathogen efficacy and diminishes SIRS (5).

### Other mtDAMPs

TFAM is abundant in the mitochondria and plays a key role in stabilizing the mtDNA structure and protecting mitochondrial function (13). TFAM damage has a dual role in the inflammatory response. On the one hand, TFAM damage disrupts mtDNA stability, leading to mtDNA escape and inflammatory response through multiple signaling pathways (53). On the other hand, TFAM, as a DAMP, enhances the secretion of proinflammatory cytokines (54).

mtRNA, synthesized through mtDNA transcription, can also lead to inflammation because of abnormal accumulation and release. Mitochondrial ssRNA can activate TLR8/MyD88 signaling pathway (55). After recognition by RIG-I, dsRNA activates NF-κB and IRF3/7 through MAVS to induce type I IFN expression (56). However, dsRNA cannot activate the type I IFN response in all cells, such as human islet β-cells (57).

CL is a crucial phospholipid in the bacterial membrane and mitochondrial inner membrane and plays a pivotal role in maintaining the electron transport chain, mitophagy, and apoptosis (58). Chen et al. discovered that the loss of Sam50 (which connects the mitochondrial inner and outer membrane) led to CL externalization. This causes mitochondrial membrane remodeling, mtDNA aggregation, and release through Bax/Bak mitochondrial pore, mtDNA/cGAS/STING signaling pathway activation, and hepatitis damage (59).

Extracellular ATP (eATP) induces the generation and release of IL-1β, IL-6, and TNF-α by activating phospholipase A2/D, MAPK, NF-κB, and other pathways (60). In the mouse model of acute pancreatitis, the levels of eATP increased and promoted proinflammatory cytokine production and induced SIRS by activating MAPK and NF-κB signaling pathways (61).

Cytochrome *c* is released from damaged cells into the extracellular space and acts as DAMP to trigger the inflammatory response. Wenzel et al. discovered that cytochrome *c* induced the inflammatory activation of microglia through the TLR4 signaling pathway (62). Moreover, Pullerits et al. discovered that extracellular cytochrome *c* triggered neutrophil-mediated and monocyte-mediated inflammation through the NF-κB signaling pathway (63).

### Interaction of mtDAMPs

Multiple signaling pathways of mtDAMPs that mediate inflammatory response interact with each other.

MtDAMPs mediate proinflammatory cytokine release and form positive feedback, leading to further release of mtDAMPs from the mitochondria and exacerbation of the inflammatory response. Aarreberg et al. demonstrated that exogenous IL-1β promotes mitochondrial aggregation in bystander cells, such as fibroblasts and epithelial cells, lowers mitochondrial membrane potential, and induces mtDNA release, thereby activating the cGAS/STING-dependent type I IFN response (64). Willemsen et al. discovered that long-term TNF stimulation also triggers the release of mtDNA and induces type I IFN response through cGAS/STING (65). In diABZIP (a kind of STING agonist)-administered mice, STING activation-induced cell death and mtDNA release, thereby activating the cGAS/STING signaling pathway (66). The excessive activation of STING amplifies the inflammatory cycle (67).

## Organ-level evaluation of trauma-related SIRS induced by mtDAMPs

Post-traumatic tissue damage leads to mtDAMP release from the mitochondria, which promotes inflammatory cytokine release through a variety of signaling pathways and mediates inflammatory response. Clinical studies have demonstrated that the plasma mtDNA levels of trauma patients were significantly higher than those of healthy individuals (10, 67); the levels continued to increase 24 h after injury (10), indicating that plasma mtDNA has a moderate discriminative power in predicting the risk of SIRS after trauma (68). In addition, the plasma mtFP levels of trauma patients with SIRS and sepsis were higher than those of controls (69). Hu et al. claimed that plasma mtDNA concentration was remarkably high in patients with intraperitoneal infection and MODS; moreover, the baseline plasma mtDNA concentration at admission could effectively predict their prognosis (70). Martinez-Quinones et al. evaluated critical patients who had undergone open laparotomy and discovered that peritoneal lavage reduced the level of mtDAMPs in ascites (71). They proposed that increasing the frequency of peritoneal lavage may decrease systemic absorption of mtDAMPs, thereby reducing the risk of aseptic SIRS (71).

One of the features of the SIRS response is a widespread inflammatory response, defined in part by immune cell activation and the production of proinflammatory cytokines. In-depth studies have demonstrated that mtDAMPs including mtDNA/mtFPs can activate innate immune cells such as antigen-presenting cells, macrophages, neutrophils, and dendritic cells play crucial roles in recognizing, phagocytosing, and releasing inflammatory mediators (8). For example, via PRRs activating antigen-presenting cells and neutrophils, the production of local ROS, cytokines, chemokines, MMPs, and NETs increased (8, 72–74). Moreover, injection of DAMPs into rodents has been shown to be associated with organ damage, while patients who have high levels of mtDNA in their circulation when sampled 2 h post-injury are at higher risk of developing MODS (75). Therefore, scavenging DAMPs may help alleviate the proinflammatory response triggered by DAMPs. For example, Lee et al. have demonstrated that nucleic acid scavenging microfiber meshes represent an effective strategy to inhibit trauma-induced inflammation and thrombosis *in vitro* and *in vivo* (76). Moreover, Aswani et al. have shown *in vitro* that the use of nucleic acid scavenging polymers, for example, hexadimethrine bromide (HDMBr), can reduce circulating mtDAMP levels and reduce the severity of organ injury in rat hemorrhagic shock models (75).

SIRS often involves multiple organ injuries and inflammation such as acute lung injury (ALI), acute respiratory distress syndrome (ARDS), acute kidney injury (AKI), and traumatic brain injury (TBI) (8). The following describes the latest research on mtDAMPs in trauma-induced SIRS at the organ level. The mechanisms and therapeutic targets of mtDAMPs in various organs of trauma-induced SIRS are also summarized in **Tables 1**, **2**.

**Table 1:** Mechanisms of post-trauma organ inflammation induced by mtDAMPs.

Organ	Type of inflammation	Mechanism
Lung	Trauma-induced ARDS	Elevated plasma mtDNA levels may predict the risk of ARDS in response to distal injury (77, 78).
	Sepsis-related ARDS	MtOGG1 repairs and inhibits ox-mtDNA release, alleviating lung neutrophils and macrophage infiltration (38).
	Sepsis-related ALI	MtFPs aggravate lung fluid imbalance in ALI through FPR1 signal (79).
	Burn-induced ALI	Elevated plasma mtDNA levels may enhance neutrophil infiltration and ALI after burn through cGAS/STING and NLRP3 signaling pathways (80, 81).
	Invasive tracheal intubation-induced tracheitis	Epithelial cell injury activates neutrophils and releases mtDNA, thereby activating TLR9 signaling pathway (82).
	Mechanical ventilation-induced lung inflammation	MtDNA is released and activates TLR9 (83), and mtFPs synergize IL-1β to promote neutrophil chemotaxis (84). PINK1-dependent mitophagy induces mtDNA release, activates mtDNA downstream signaling pathway, and mediates inflammatory response (85).
Kidney	Trauma-induced AKI	There is a significant temporal correlation between post-traumatic circulating mtDNA level and severity of AKI (86).
	IR-AKI	RIP3 promotes mitochondrial degradation and mtDNA release, activating cGAS/STING (87). MtROS inhibited renal TFAM transcription and promoted its degradation, resulting in mtDNA damage (88). Kidney PGAM5 is upregulated to promote Bax-dependent mtDNA release and initiate cGAS/STING signaling pathway (89).
Cardiovascular	Hemorrhagic shock	Increased plasma mtFPs activate FPR, leading to NO release and severe hypotension (90). Increased mtDNA release and ROS contents in myocardial tissues activate the systemic inflammatory response (14).
	Dysfunction of blood vessel	MtDNA/cGAS/STING signaling pathway inhibits endothelial proliferation and vascular repair by downregulating YAP signal (91). MtDAMPs influence endothelial cells and neutrophils through diverse signals, which can promote the adherence and interactions of neutrophils to endothelial cells, consequently elevating systemic endothelial permeability (92).
	Heart operation	Early postoperative plasma mtDNA level is associated with postoperative SIRS and multi-organ failure (93). The plasma mtDNA level of patients undergoing MiECC is lower than that during CPB surgery and is positively correlated with postoperative myocardial injury (94). MtDAMPs such as TFAM and cytochrome *c* elevated in venous grafts after CABG surgery (95).
Brain	TBI	Ccf-mtDNA elevated in CSF and serum within 48 h after acute brain injury (96). Ccf-mtDNA level in CSF is correlated with injury severity and has a stronger predictive effect on neuronal injury and inflammation after TBI (96, 97). NLRP3 inflammasome of microglia was activated, releasing inflammatory cytokines such as IL-1β and IL-18 (98), and NLRP3 peak is associated with poor prognosis (99). Gasdermin D is a downstream factor of NLRP3 inflammasome activation (100). MaxiK expression was significantly increased in the cerebral cortex, which may activate NLRP3 inflammasome by promoting K^+^ transport (94). STING/NLRP3 signaling pathway was involved in neuroinflammation after TBI (101). MtDNA/cGAS/STING and its mediated type I IFN response play an important role in the neuroinflammatory response after TBI, mainly in microglia (102–104). STING expression was significant upregulation in late traumatic human brain samples (105). Compared with young mice, aged mice showed greater activation of cGAS/STING and type I IFN (103, 104). NLRX1 restricts the activation of cGAS/STING and the overexpression of type I IFN after TBI (102).
Bone and muscle	Orthopedic trauma operation	There is a correlation between preoperative mtDNA level and post-traumatic time, and also a correlation between the duration and magnitude of surgical intervention and postoperative mtDNA concentration (103).
	Cartilage damage	Synovial mtDNA levels increased (17). Extracellular mtDNA is associated with post-traumatic arthritis and is an important marker of early cartilage damage (106).
Liver	Liver transplantation	Plasma mtDAMPs increase during liver transplantation, which is relevant to the prognosis (107).
	Burn-induced live injury	Liver NLRP3 inflammasome activates Kupffer cells and releases inflammatory cytokines by recognizing ox-mtDNA (108).
Intestines	Pancreaticoduodenectomy	Circulating mtDNA level after operation is correlated with inflammatory response (109).
	IR	MtDNA derived from intestinal epithelial cells mediates proinflammatory cytokines production via TLR9 (110).

mtDNA, mitochondrial DNA; ARDS, acute respiratory failure syndrome; mtOGG1, 8-oxidative mitochondrial DNA glycosidase 1; ox-mtDNA, oxidized mitochondrial DNA; ALI, acute lung injury; mtFPs, mitochondrial formyl peptide; FPR1, formyl peptide receptor 1; AKI, acute kidney injury; IR, ischemia–reperfusion; TLR9, Toll-like receptor 9; PINK1, PTEN-induced putative kinase 1; RIP3, receptor-interacting protein 3; PGAM5, phosphoglycerate mutase 5; SIRS, systemic inflammatory syndrome; CPB, cardiopulmonary bypass; MiECC, minimized extracorporeal circulation; CABG, coronary artery bypass grafting; TBI, traumatic brain injury; ccf-mtDNA, circulating cell-free mtDNA; CSF, cerebrospinal fluid; NLRX1, NLR containing X1; mtDAMPs, mitochondrial damage-associated molecular patterns; TFAM, mitochondrial transcription factor A.

**Table 2:** Therapeutic measures targeting mtDAMPs and the signaling pathways.

Organ	Drug/targets	Role and signaling pathways
Lung	Cyclosporine-A	Reduce lung mtDNA release and oxidative stress in burn and sepsis (86, 111).
	Metformin	Block new mtDNA synthesis, thereby blocking NLRP3 activation and improving ARDS (38).
	EGCG	Clear mtROS and ox-mtDNA, inhibit NLRP3 activation, and protect lung injury caused by acute pancreatitis (112)
	Tanreqing	Alleviate sepsis-related ALI by inhibiting mtDNA/cGAS/STING signaling pathway (113).
	PSPAs	Promote Parkin-dependent mitophagy, reduce the release of mtROS and mtDNA, and inhibit NLRP3 activation (114).
	Suhuang	Maintain mitochondrial homeostasis, reduce mtROS overproduction and mtDNA release, downregulate MMP9 expression, and inhibit NF-κB and NLRP3 signaling pathways (115).
	Corticosteroids	Inhibit NF-κB and mtROS-dependent NLRP3 activation (116).
	MSC-EVs	Regulate alveolar epithelial-capillary barrier integrity and improve mitochondrial oxidative phosphorylation of macrophages through mitochondrial transfer (117–119).
Kidney	SS31 peptide	Targeted inhibit mtROS/NLRP3 pathway to improve mitochondrial oxidative stress in kidney (120).
	MEC-EVs	Restore the stability of TFAM and TFAM–mtDNA complexes, thereby reversing mtDNA depletion in damaged kidney cells (121).
Cardiovascular	TLR9 antagonists	Block TLR9 downstream signaling pathway, thereby significantly reducing IL-6 expression (122).
	Sulforaphane	Reduce mtDNA release (122).
	SkQ1	Protect the ultrastructure of rat myocardial mitochondria, reduce mtDNA release and the ROS content, and thus reduce inflammation (14).
Brain	NLRP3 inhibitors (MCC950, gastrodin, ACT001, oridonin, and parthenolide)	Inhibit the expression and assembly of NLRP3 inflammasome components, reduce the secretion of IL-1β and IL-8, effectively alleviate the inflammatory response and BBB damage after TBI, and play a protective role on neurons (123–127).
	LIFUS	Inhibit the activation of NF-κB and NLRP3 inflammasome after TBI by promoting the expression of orexin-A and orexin receptor 1 (101).
	Let-7i	Intranasal injection of let-7i can inhibit the expression of STING and reduce neuronal apoptosis after TBI (128).
Bone and skeletal muscle	SS31 peptide	Reduce mtDNA to baseline level (17).
	CoQ10	Decrease mtDNA, inhibit the expression of ASC and NLRP3, and reduce the levels of IL-1α, IL-1β, and IFN-γ, thereby alleviating the systemic inflammatory response after skeletal muscle burn (129).
Intestines	ACA	Inhibit mtROS production and mtDNA oxidation to reduce NLRP3 inflammasome activation and alleviate colitis (130).
MODS	nucleic acid scavenging (microfiber meshes, HDMBr)	Nucleic acid scavenging microfiber inhibits trauma-induced inflammation and thrombosis *in vitro* and *in vivo* (76). Nucleic acid scavenging polymers hexadimethrine bromide (HDMBr) can reduce circulating mtDAMP levels and reduce the severity of organ injury in rat hemorrhagic shock models (75).

EGCG, epigallocatechin-3-gallate; PSPAs, purple sweet potato anthocyanins; MSC-EVs, mesenchymal stem cell-extracellular vesicles; SS31, D-Arg-Dmt-Lys-Phe-NH2; ACA, 1′-acetoxychavicol acetate; BBB, blood–brain barrier; TBI, traumatic brain injury; LIFUS, low-intensity focused ultrasound; mtDNA, mitochondrial DNA; ARDS, acute respiratory distress syndrome.

### Lung

ARDS is an acute inflammatory lung injury. Clinical studies have discovered that plasma mtDNA levels correlate with ARDS severity in trauma patients and can also predict the risk of ARDS during distal injury (77, 78).

MtFPs aggravate lung fluid imbalance through the FPR1 signaling pathway (79). *In vivo* experiments of burn-induced ALI mice revealed that elevated plasma mtDNA levels may enhance neutrophil infiltration and post-burn ALI through cGAS/STING and NLRP3 signaling pathways (80, 81).

Acute tracheitis is often secondary to invasive endotracheal intubation because of epithelial cell damage. This activates neutrophils and mtDNA release, which mediate proinflammatory cytokine secretion through the mtDNA/TLR9/NF-κB signaling pathway (82). In addition, mechanical ventilation can lead to excessive lung traction and mechanical damage, resulting in the activation of the TLR9/MyD88/NF-κB signaling pathway (83). MtFPs cooperate with IL-1β to promote neutrophil chemotaxis (84), thereby aggravating inflammation and lung injury. In the cyclic stretching cell culture model constructed by Jing et al., the overexpression of PTEN-induced putative kinase 1 (PINK1) in lung epithelial cells exacerbated stretch-induced inflammatory response (85) through PINK1-dependent mitophagy to induce mtDNA release (85). Therefore, the inhibition of mitophagy and mtDNA-mediated TLR9/MyD88/NF-κB signaling pathway may be a potential therapeutic approach for lung injury caused by mechanical ventilation (85). Xian et al. discovered that 8-oxoguanine DNA glycosylase 1 (mtOGG1), an mtDNA base excision repair enzyme, repaired ox-mtDNA and inhibited its release, thereby alleviating pulmonary vascular endothelial injury and infiltration of neutrophil and macrophage; this provided resistance to LPS-induced ARDS in mtOGG1 transgenic mice (38).

The inhibiting of mtDAMP synthesis and release, blockage of downstream signaling pathways of mtDAMPs, and maintenance of mitochondrial homeostasis may control pulmonary inflammation. Cyclosporine-A attenuates oxidative stress and mtDNA release in lung tissue in a dose-dependent manner, thereby exerting a protective effect on both burn-induced ALI and LPS-induced ALI (86, 111). Xian et al. discovered that metformin ameliorated ARDS by inhibiting mtDNA synthesis and blocking NLRP3 inflammasome activation (43). Epigallocatechin-3-gallate (EGCG) may inhibit NLRP3 inflammasome activation by clearing mtROS and ox-mtDNA, thereby protecting against lung injury caused by acute pancreatitis (112). In a study, Tanreqing significantly alleviated LPS-induced ALI by inhibiting the mtDNA/cGAS/STING signaling pathway (113). Dong et al. reported that purple sweet potato anthocyanins (PSPAs) inhibited NLRP3 inflammasome activation by promoting parkin-dependent mitophagy and reducing the release of mtROS and mtDNA; this resulted in reduced lung inflammation and mortality of *Klebsiella pneumoniae*-infected mice (114). Suhuang exhibited a positive effect on mitochondrial homeostasis in ALI mice by reducing mtROS overproduction and mtDNA release, downregulating MMP9 expression, and inhibiting NF-κB and NLRP3 inflammasome activation (115). Corticosteroids protect against inflammation response and ALI by inhibiting the NF-κB signaling pathway and mtROS-dependent NLRP3 inflammasome activation (116). Mesenchymal stem cell-extracellular vesicles (MSC-EVs) regulate alveolar epithelial–capillary barrier integrity through mitochondrial transfer; this restores metabolic and immune homeostasis of airway macrophages and thereby reduces the release of alveolar mtDNA, which effectively alleviates lung inflammation and improves organ function (117–119).

### Kidney

In critically injured patients, the continuous monitoring of circulating mtDNA levels within 48 h after trauma has a significant temporal correlation with AKI (131). AKI is frequent among patients with severe burns and is associated with high mortality (132). Although the incidence of AKI is low, late AKI is severe and is a poor prognostic factor for severe burns (132).

According to Feng et al., receptor-interacting protein 3 (RIP3) promotes mitochondrial degradation and mtDNA release, which activates the cGAS/STING signaling pathway and exacerbates inflammation and kidney injury after renal IR (87). Zhao et al. noted renal TFAM deficiency and mtDNA damage in patients with IR-AKI; in the mouse model, mtROS disrupted TFAM and mtDNA homeostasis by inhibiting renal TFAM transcription and promoting its degradation, thereby driving mtDNA release and renal inflammatory response (88). Li et al. discovered that phosphoglycerate mutase 5 (PGAM5) was upregulated in the kidneys of AKI human biopsy samples and mouse models; the upregulation promoted Bax-dependent mtDNA release and initiated the mtDNA/cGAS/STING signaling pathway (89).

In a cisplatin-induced AKI mouse model, mitochondrial-targeted therapy with SS31 peptide (D-ARG-DMT-lys-Ph-NH2, a mitochondrial targeting antioxidant) improved renal oxidative stress by inhibiting the mtROS/NLRP3 signaling pathway (120). In addition to lung inflammation alleviation, MSC-EVs play an active role in kidney inflammation. Zhao et al. reported that the application of MSC-EVs restored the stability of TFAM and TFAM–mtDNA complexes, thereby reversing damage caused by mitochondrial oxidative phosphorylation in renal tubular cells and alleviating kidney inflammation (121). However, intravenous injection of MSC-EVs into mice with weakened TFAM expression had poor efficacy, and TFAM overexpression had better efficacy (121). These results suggest that MSC-EVs are a promising nanotherapy for diseases with mitochondrial damage; the TFAM signaling pathway is essential for maintaining mitochondrial regenerative capacity (121).

AKI can damage distal organs such as the lung. Hepokoski et al. discovered that mitochondrial dysfunction occurred in the lungs and systemic circulation of IR-AKI mice, leading to an increase in extracellular mtDNA and TFAM levels and enhanced infiltration of pulmonary neutrophils (133). Intraperitoneal injection of renal-derived mtDAMPs resulted in metabolomic changes caused by lung mitochondrial dysfunction *in vivo* (133). Therefore, mitochondrial function and mtDAMPs may be potential therapeutic targets for preventing AKI-related lung injury (133).

### Cardiovascular

MtFPs mediate inflammation through FPR activation, also dilate resistant arteries, and induce vascular endothelial cell dysfunction in a blood-dependent manner (90). Specifically, the collapse of blood vessels is the primary pathophysiology feature of the sepsis-like syndrome. F-MIT does not affect the arteries’ relaxation induced by acetylcholine. However, arteries incubated in blood containing F-MIT or blood from rats treated with F-MIT show reduced relaxation compared to their respective control groups. These findings indicate that F-MIT induces blood-dependent endothelial dysfunction (90). In a rat model of hemorrhagic shock, mtFPs lead to NO release and severe hypotension through FPR, and elevated plasma mtFP levels were associated with aseptic trauma-induced ALI (90). Moreover, the activation of the mtDNA/cGAS/STING signaling pathway inhibits endothelial proliferation and vascular repair by downregulation of the YAP signaling pathway (91). Therefore, mtFPs may serve as a bridge between trauma, SIRS, and cardiovascular failure (90). Moreover, the release of mtDAMPs into the bloodstream can occur through various mechanisms, causing cellular damage and resulting in increased pathological endothelial permeability. Sun et al. demonstrated that the mitochondria contain numerous DAMP motifs capable of influencing endothelial cells and neutrophils through diverse signals, which can promote the adherence and interactions of neutrophils to endothelial cells, consequently elevating systemic endothelial permeability (92). Recent research by our team has also identified myocardial mitochondrial structure disruption in the rat model of hemorrhagic shock (HS), resulting in increased mtDNA release and ROS contents, which in turn activates the systemic inflammatory response (131). More importantly, we found the mitochondrial antioxidant SkQ1 can protect myocardial mitochondria, improve the ultrastructure of rat myocardial mitochondria, reduce mtDNA release and the ROS content, and thus reduce inflammation (14). Cardiopulmonary bypass, surgical trauma, and ischemia–reperfusion injury stimulate a systemic inflammatory response in cardiac surgery. Early postoperative plasma mtDNA level is a predictive marker for SIRS and multi-organ failure in patients undergoing cardiac surgery (93). The elevated plasma mtDNA level during cardiopulmonary bypass (CPB) surgery may be involved in SIRS pathogenesis and the related postoperative inflammatory events (such as postoperative atrial fibrillation and infection) (67). Compared with traditional CPB, minimally invasive extracorporeal circulation (MiECC) results in a lower plasma mtDNA level, positively correlated with CPB duration and postoperative myocardial injury (94). Naase et al. further confirmed that mtDNA-mediated inflammation through TLR9 and TLR9 antagonist administration significantly reduced IL-6 expression (122). The antioxidant sulforaphane reduces mtDNA release and may have a potential therapeutic role in stimulating the systemic inflammatory response in cardiac surgery (122).

### Brain

TBI can damage the blood–brain barrier (BBB), which can lead to neuroinflammation. This inflammation can be caused by mtDAMPs, which trigger the release of proinflammatory cytokines and polarize microglia/macrophages toward an M1-like phenotype (134). A series of previous studies have proved that the levels of IL-1β, IL-8, TNF-α, and other cytokines in serum and cerebrospinal fluid (CSF) of TBI patients increased, which were correlated with the degree of tissue damage; IL-1β might especially be an independent prognostic factor after TBI (135, 136). The increased expression of these cytokines is related to TBI-mediated mtDNA release (121). Within 48 h after TBI, circulating cell-free mtDNA (ccf-mtDNA) levels in both CSF and serum are elevated (96). Moreover, compared with serum, ccf-mtDNA level in CSF is correlated with injury severity and inflammatory cytokine response and has a more robust predictive effect on neuronal injury and inflammation after TBI (96, 97).

The circulating mtDNA was first identified as the mtDAMP activated by NLRP3 (10) and a crucial component of the NLRP3 activation pathway (37, 42). Moreover, mitochondrial dysfunction-induced mtDNA and mtROS release after TBI could serve as stimuli regulating the NLRP3 inflammasome downstream by transcriptional or post-translational modifications (137). Therefore, we further elucidate the role of the NLRP3 inflammasome in TBI. NLRP3 inflammasome of microglia is involved in the development of neuroinflammation after TBI. Liu et al. showed for the first time that TBI induced upregulation of NLRP3-related genes and proteins in the mouse cerebral cortex, activating NLRP3 inflammasome assembly and releasing inflammatory cytokines such as IL-1β and IL-18 (98). The concentration of NLRP3 in the CSF of infants with TBI changes significantly with time after trauma, and the NLRP3 peak is associated with poor prognosis (99). It is worth noting that NLRP3 knockdown does not lead to changes in IL-1β production, but some markers of microglia and astrocytes will be overexpressed, increasing cytokine levels (138). As a downstream factor of NLRP3, the expression of GSDMD is also blocked (100). According to RNA sequencing, both GSDMD knockdown and NLRP3 knockdown reversed the expression of genes related to neuroinflammation after TBI (100). MaxiK channel, also known as large-conductance Ca^2+^-activated K^+^ channels, big K^+^ (BK) channels, is important for K^+^ transport. MaxiK channels are important in a variety of physiological functions, including regulation of neuronal firing, endocrine cell secretion, smooth muscle tone, and cellular proliferation and migration (139, 140). In explosion-induced TBI, MaxiK significantly increased in the cerebral cortex, which may activate NLRP3 inflammasome by promoting K^+^ transport. Blocking this channel can effectively inhibit NLRP3 activation and neuroinflammatory response (139). The STING/NLRP3 signaling pathway has also been shown to be involved in neuroinflammation, and the NLRP3-mediated inflammatory response can be partially inhibited by blocking the expression of STING (101, 136).

NLRP3 inhibitors can restrain the expression and assembly of NLRP3 inflammasome components and reduce the secretion of caspase-1-activated IL-1β and IL-8, thereby effectively alleviating the inflammatory response and BBB damage after TBI, playing a protective role on neurons (123, 126, 127). As a specific inhibitor of NLRP3, the neuroprotective effect of MCC950 depends on the presence of microglia and is limited to the first 6 h after TBI (123). MCC950 combined with rapamycin treatment further enhances neuroprotection after TBI through rapamycin-mediated mitochondrial phagocytosis (141). In addition to inhibiting the expression of NLRP3, oridonin extracted from Chinese herbal medicine can also improve mitochondrial function by enhancing the activation of the Nrf2 signal and reducing the number of degenerated neurons and the volume of cortical lesions (124). Parthenolide treatment simultaneously suppresses STAT3/NF-κB and NLRP3 inflammasomes, thereby inhibiting microglial activation, alleviating neurological deficits, and improving memory and learning in TBI mice (125). In addition to drug therapy, low-intensity focused ultrasound (LIFUS), as a novel treatment for neurological diseases, significantly inhibited the activation of NF-κB and NLRP3 inflammasome after TBI by promoting the expression of orexin-A and orexin receptor 1 (142).

Previous studies have demonstrated that STING mRNA is significantly upregulated in post-mortem human TBI brain samples (105). In recent years, many experimental studies have shown that mtDNA/cGAS/STING and its mediated type I IFN response play an important role in the neuroinflammatory response after TBI, and microglia are the main cell type expressing cGAS and STING in the brain (102–104). STING activation is also age-related. Compared with young mice, aged mice showed greater activation of cGAS/STING and significantly upregulated type I IFN response (103, 104). Fritsch et al. found that NLR containing X1 (NLRX1) could limit the activation of cGAS/STING after brain injury and inhibit the overexpression of type I IFN (102). Let-7i is the upstream signal of STING, whose expression level decreases in TBI mice (128). Intranasal injection of let-7i helps to inhibit the expression of STING, reduce neuronal apoptosis, and improve the cognitive function of mice (128).

### Bone and muscle

A study of heterogeneous orthopedic trauma patients emphasizes the sustained presence of predominantly mtDNA in the plasma of trauma patients following surgical intervention. The correlation between the degree and timing of surgery and mtDNA concentration suggests that mtDNA may serve as a potential marker for a postoperative secondary strike and post-traumatic complications (143). Seewald et al. demonstrated for the first time the relationship between extracellular mtDNA and post-traumatic osteoarthritis (PTOA) (106). In another study in horses, mechanically induced cartilage injury resulted in increased synovial mtDNA concentration either through the selective release of mtDNA from living cells or through cell death or rupture; however, treatment with SS31 peptide effectively reduced mtDNA levels to the baseline level (17). These results suggest that synovial mtDNA concentration is a non-invasive method to detect cartilage dysfunction and acute cartilage injury; moreover, mitochondrial protective drugs may be a novel PTOA prevention and treatment strategy (17).

In smooth muscle, Thankam et al. found that TFAM, cytochrome *c*, and other mtDAMPs were elevated in venous grafts after coronary artery bypass grafting (CABG), which was related to the increase of ROS content in hypoxic smooth muscle and the damage of membrane integrity, leading to graft failure (95). In mice with skeletal muscle burn, CoQ10 treatment decreased mtDNA level, inhibited the expression of ASC and NLRP3, and reduced the levels of IL-1α, IL-1β, and IFN-γ, thereby alleviating the systemic inflammatory response after burn (129).

### Liver

In a retrospective study on patients undergoing a liver transplant, Nagakawa et al. discovered that the measurement of plasma mtDAMPs might predict the post-transplantation recovery of the patients (107). In burn and delayed resuscitation experiments in rats, liver NLRP3 inflammasomes activated Kupffer cells by recognizing ox-mtDNA, releasing inflammatory cytokines, and causing liver injury (108).

### Intestines

The circulating mtDNA level after pancreaticoduodenectomy is associated with inflammatory responses and may be used as an early marker of the postoperative disease course (109). During intestinal IR, mtDNA derived from intestinal epithelial cells mediates proinflammatory cytokine generation through the TLR9 signaling pathway, which exacerbates acute inflammatory response (110). Sok et al. constructed mouse models of peritonitis and colitis and demonstrated that 1′-acetoxychavicol acetate (ACA; a natural compound in the rhizome of tropical ginger) inhibited mtROS production and mtDNA oxidation to reduce NLRP3 inflammasome activation, thereby alleviating the colitis in mice (130).

## Conclusion

**Figure 1:**
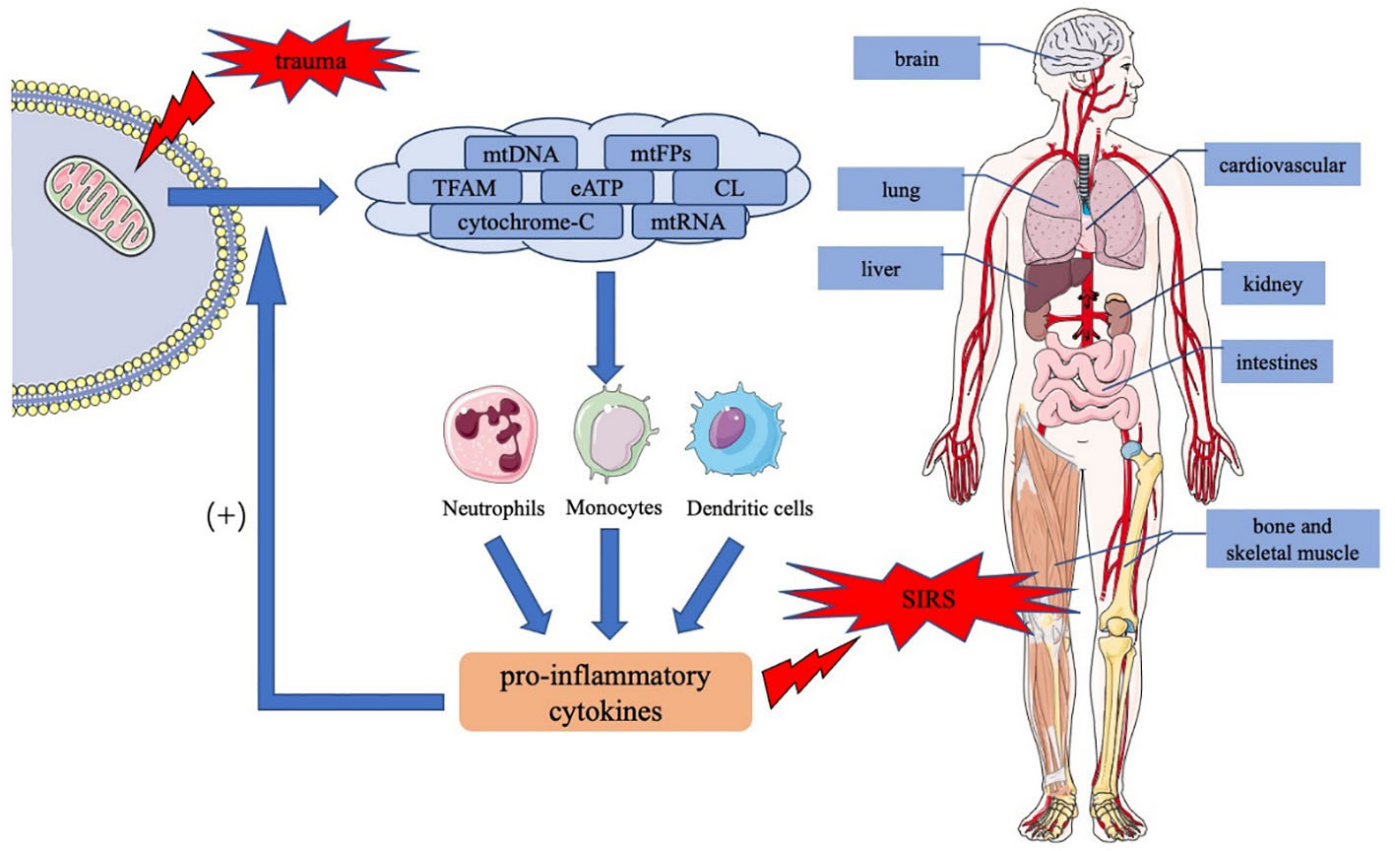
The role of mtDAMPs in the trauma-induced systemic inflammatory response syndrome. Trauma causes tissue damage, resulting in the release of mtDAMPs including mtDNA, mtFPs, TFAM, eATP, CL, cytochrome *c*, and mtRNA, in the injured mitochondria. MtDAMPs activate downstream signaling pathways in immune cells, such as neutrophils, monocytes, and dendritic cells, thereby inducing the production and release of proinflammatory cytokines. In addition, proinflammatory cytokines act positively on normal cells, increasing mtDAMP release and exacerbating inflammatory response. An uncontrolled inflammatory response causes SIRS and the dysfunction of multiple organs throughout the body. mtDAMPs, mitochondrial damage-associated molecular patterns; mtDNA, mitochondrial DNA; mtFPs, mitochondrial formyl peptides; TFAM, mitochondrial transcription factor A; CL, cardiolipin; eATP, extracellular ATP; mtRNA, mitochondrial RNA; SIRS, systemic inflammatory response syndrome.

Post-trauma tissue damage releases mtDAMPs from the mitochondria into the cytoplasm or the extracellular space, leading to proinflammatory cytokine release and immune cell activation through a series of signaling pathways. MtDAMP-induced inflammatory reaction protects the body; however, excessive inflammation damages organ function and causes SIRS and MODS, which are poor prognostic factors for trauma patients. **Figure 1** summarizes the role of mtDAMPs in the trauma-induced systemic inflammatory response syndrome. For the optimal management of trauma-induced SIRS, further clinical studies on mtDAMPs are required such as the quantification of mtDAMPs levels in different trauma patients, the correlation of mtDAMPs with trauma injury severity or clinical outcomes, the potential intervention strategies targeting mtDAMPs to reduce traumatic SIRS, and the effectiveness and safety assessments. Such studies may lead to the usage of mtDAMPs, especially mtDNA, as biomarkers for predicting the course and prognosis of SIRS in trauma patients. Drugs targeting mtDAMPs and maintaining mitochondrial homeostasis are promising therapeutic strategies.

## Author contributions

TW and JY designed the project; JY and XH wrote the paper; ZW, RL, LG and MZ performed literature search and revision; and all authors participated in the data analysis and interpretation. All authors contributed to the article and approved the submitted version.
